# Exploring long covid care pathways in the UK NHS: a qualitative study of healthcare professionals’ experiences

**DOI:** 10.1136/bmjopen-2026-120704

**Published:** 2026-09-15

**Authors:** Lanre Daodu, Yogini Raste, Judith E Allgrove, Francesca Arrigoni, Reem Kayyali

**Affiliations:** 1 Kingston University London, Kingston Upon Thames, Surrey, UK; 2 Croydon University Hospital, London, UK; 3 Bournemouth University, Poole, UK

**Keywords:** QUALITATIVE RESEARCH, Post-Acute COVID-19 Syndrome, Health Workforce, Health Services, General Practice

## Abstract

**Abstract:**

**Objectives:**

To explore the operational reality of the long covid care pathway from the perspective of National Health Service (NHS) professionals, addressing the gap between national guidelines and frontline implementation.

**Design:**

A qualitative study using single-timepoint, semi-structured interviews. Data were collected between July and October 2024. Data were analysed using reflexive thematic analysis.

**Setting:**

Primary and secondary NHS care settings in a diverse South London Borough (Croydon), UK.

**Participants:**

12 NHS healthcare professionals managing adult patients, comprising general practitioners (GPs, n=5), respiratory consultants (n=2), specialist nurses (n=3), a respiratory physiotherapist (n=1) and a clinical psychologist (n=1).

**Results:**

The operational reality of long covid care was constructed around four central themes. First, lacking definitive biomarkers, clinicians navigated diagnostic uncertainty using pragmatic, exclusionary testing and subjective severity assessments. Second, GPs acted as administrative linchpins, compelled to triage patients into fragmented, symptom-driven specialist silos. Third, treatment demonstrated therapeutic pragmatism, relying heavily on non-pharmacological rehabilitation rather than medicalised cures. Finally, variable monitoring practices across secondary services created a post-discharge ‘continuity gap’, placing the burden of re-initiating care on the patient. These systemic frictions were synthesised into an eight-step operational care pathway map.

**Conclusions:**

While national guidelines provide best practices, this study uniquely captures the systemic bottlenecks that occur when operationalising these standards locally. The mapped pathway reveals a pressing need for the standardisation of functional assessment tools, genuinely integrated interdisciplinary clinics and robust post-discharge tracking to mitigate the burden of coordination on primary care.

**STRENGTHS AND LIMITATIONS OF THIS STUDY**

- This study uses a qualitative, single-timepoint interview design to capture the complex operational realities of frontline staff, moving beyond superficial service audits to provide deep contextual insights.
- The purposive, multidisciplinary sample, spanning primary care, secondary respiratory medicine and allied health rehabilitation, prevents the data from being skewed by a single professional silo, enabling a comprehensive mapping of the complete care pathway.
- High methodological and interpretive rigour is maintained through the application of Braun and Clarke’s reflexive thematic analysis and strict adherence to the Consolidated Criteria for Reporting Qualitative Research (COREQ) reporting guidelines.
- A methodological limitation is the geographic focus on a single diverse London borough; while sample size sufficiency was rigorously justified using the information power framework, specific structural bottlenecks may differ across other National Health Service Trusts.
- The study design focuses exclusively on the provider perspective and relies on self-selecting participants, meaning recruitment bias is possible and the direct lived experience of patients navigating the system is not captured.

## Introduction

 Long covid emerges as a complex, multisystem condition requiring coordinated, multidisciplinary team (MDT) management. The physiological complexity of the condition necessitates a collaborative effort, requiring a workforce of healthcare professionals from various disciplines to ensure patients receive comprehensive, personalised care. Globally, health and research institutions have implemented strategies to elucidate optimal care protocols, including research into evidence-based interventions to alleviate symptoms and enhance quality of life.^1–3^ However, translating biomedical research into practical clinical use is often lengthy and inconsistent.^4^ This translational gap can result in significant delays, leaving patients without access to the most beneficial and up-to-date care.^4^ Clinical Care Pathways (CCPs) play a crucial role in bridging this gap between research and practice.^5^ CCPs are used globally as essential instruments of clinical governance.^6 7^ CCPs aim to outline the most effective care trajectory for complex clinical conditions, reducing variation in practice and optimising resource allocation.^7^

Evidence from a scoping review indicates that primary care is best placed to manage the condition, with complex cases referred to specialist clinics and single-symptom cases directed towards targeted assessments.^8^ Structured pathway documents are recommended to facilitate effective communication between healthcare professionals and patients and support self-management.^8–10^ European randomised controlled trials have previously demonstrated that CCPs reduce clinical variation and enhance MDT work in chronic conditions.^11–13^ However, evidence suggests that implementing these pathways for long covid remains challenging. A UK-based study involving 2500 patients found that access to care was highly variable.^14^ While the majority accessed care through their general practitioner (GP), only 50% of long covid patients felt their condition was adequately recognised. The study reported that GPs referred between 71% and 76% of patients to secondary care specialists; however, only 35% of these referrals were to dedicated post-COVID clinics, with the remainder sent to symptom-specific silos, resulting in fragmented care.^14^

Significant progress has been made in defining best-practice models for long covid care, notably through large-scale initiatives such as the National Institute for Health and Care Research-funded LOCOMOTION and STIMULATE-ICP studies, which established ‘gold standard’ pathways for assessment and rehabilitation.^15 16^ However, these foundational studies mapped care pathways between 2021 and 2023, during a period of dedicated, ring-fenced national funding for long covid services. As these initial funding mechanisms have shifted or ceased, the operational burden has pivoted heavily back to routine primary care. Our data, collected in mid-to-late 2024, capture this critical transition phase. Translating high-level protocols into operational reality remains a significant challenge for individual National Health Service (NHS) Trusts, particularly those adapting to the loss of dedicated pandemic-era resources. Also, establishing a local CCP for long covid is vital to prevent care fragmentation, which is associated with poorer patient outcomes and increased healthcare costs.^17^ While large-scale multi-site evaluations have successfully defined gold-standard frameworks for long covid, this study aimed to address this implementation gap by mapping the lived operational pathway within the NHS in the London Borough of Croydon. The study explored how healthcare professionals navigate operational frictions and systemic bottlenecks that arise when frontline staff attempt to translate national guidelines into local practice. The care pathway aims to improve patient outcomes, support best practice and enhance collaboration across teams.

## Methods

### Research paradigm

This study was underpinned by an interpretive-constructivist paradigm, recognising that the reality of the long covid care pathway is not a fixed entity but is socially constructed and experienced differently by various stakeholders. Ontologically, the study adopted a relativist stance, acknowledging that a GP’s reality of the pathway may differ significantly from that of a respiratory consultant. Epistemologically, a subjectivist approach was maintained, recognising that knowledge is co-constructed through the interaction between the researcher and the participant.^18^ To ensure rigour, transparency and reflexivity were maintained throughout the process, the researchers used a reflective journal, which allowed them to critically examine their own assumptions and their influence on the data.^19^ This flexible methodological framework facilitated the inductive construction of themes that captured the nuanced professional experiences of the participants rather than merely describing surface-level processes.

### Study design and setting

This study employed a qualitative design using single-timepoint, semi-structured interviews and was conducted within the NHS in the London Borough of Croydon. The London Borough of Croydon was selected as it is a densely populated and highly diverse urban area, characterised by significant variations in socioeconomic status and pockets of high deprivation, making it an ideal setting to explore the intersection of resource constraints and post-pandemic recovery. A qualitative approach was selected to explore healthcare professionals’ complex, contextualised experiences and perspectives and to capture insights regarding the ‘lived’ clinical pathway that could not be fully captured by numerical measures alone.^20^ This one-point-in-time design captured a detailed snapshot of current pathway configurations and professional perspectives on long covid care. The study focused exclusively on adult patients (≥18 years) with long covid services. This study was reported in line with the Consolidated Criteria for Reporting Qualitative Research (COREQ) 32-item checklist (online supplemental file 1).

### Participants

The study targeted NHS healthcare professionals with direct responsibility for the triage, diagnosis and management of long covid. Eligible participants met all of the following criteria: (i) at least 6 months’ experience directly managing adult patients with long covid; (ii) currently practising within an NHS-funded clinical setting (including primary care, community rehabilitation or secondary care) that served London Borough of Croydon residents; and (iii) able and willing to provide informed consent.

### Sampling and recruitment

Sampling followed principles of purposive selection and information power,^21 22^ focusing on healthcare professionals who could offer rich, experience-based insights into long covid care. To ensure diversity of perspectives rather than representativeness, participants were drawn from multiple relevant professional groups involved in the management of long covid patients. This ensured the dataset captured a wide range of operational experiences.

Eligible healthcare professionals from key disciplines were identified by local service leads and invited to participate via email, with information sheets and consent forms provided. Of the 15 approached, three declined due to workload pressures. The sample comprised 12 healthcare professionals across primary care, respiratory medicine, specialist nursing and allied health rehabilitation, ensuring broad multidisciplinary pathway coverage. Recruitment was guided by information power rather than data saturation.^22^ With a clear aim and expert participants, 12 interviews were considered sufficient to map the care pathway.^23^

### Data collection

Data collection took place from July to October 2024 to capture the operational reality of long covid services as post-pandemic transitional models became routine practice. Semi-structured interviews (online supplemental file 2) used a topic guide developed from current literature and National Institute for Health and Care Excellence guidance (NG188). Piloted with one independent GP, the final guide examined referral mechanisms, diagnostic criteria, multidisciplinary collaboration, discharge planning and local operational workarounds. To accommodate severe clinical scheduling constraints, nine interviews were conducted synchronously (face-to-face and via Microsoft Teams) in confidential, one-on-one settings. These sessions lasted 25–45 min, were audio-recorded and transcribed verbatim. To minimise the administrative burden on this severely time-poor cohort, transcripts were not returned for participant review.

The remaining three participants completed the interview asynchronously via secure email. This well-established qualitative medium^23 24^ is increasingly recommended for engaging hard-to-reach, time-poor professionals, allowing them the flexibility to reflect deeply on complex questions.^25^ To ensure analytical depth, an iterative protocol was employed: an initial prompt set was followed by two to three rounds of targeted follow-up emails to actively probe latent meanings. These multi-stage exchanges yielded a median of approximately 800 words per participant. While the absolute word count of an asynchronous email exchange is naturally lower than a spoken transcript, the data density (Information Power) was judged to be high, as participants had the opportunity to meticulously reflect on and refine their written answers.^25^

### Analytical procedure

The data were analysed using reflexive thematic analysis (RTA) following the six-phase approach outlined by Braun and Clarke,^26^ chosen for its depth and interpretive focus on systemic challenges. During phase 1 (familiarisation with the data), the lead researcher repeatedly read all transcripts, making initial and reflexive notes on operational bottlenecks. For phase 2 (generating codes), inductive, line-by-line coding was carried out in NVivo (Release 14, QSR International, CO, USA), capturing both explicit (semantic) and underlying (latent) content. To enrich interpretation, a second researcher independently coded four transcripts. In line with RTA, this was not for inter-rater reliability but to discuss and merge differing interpretations, resulting in a flexible, evolving code list.

Moving to phase 3 (constructing themes), related codes were clustered into candidate themes using NVivo’s node hierarchies and concept maps, actively generated by the researchers to reflect the clinical pathway’s operational reality. In phase 4 (reviewing themes), these candidate themes were reviewed against the coded extracts and the full dataset to ensure accuracy and comprehensiveness. Analytical memos documented decisions on theme merging or splitting, maintaining transparency in interpretation. During phase 5 (defining and naming themes), the candidate themes were refined from descriptive labels into active, interpretive concepts. Finally, in phase 6 (writing up), the analysis was woven into a cohesive narrative argument, richly supported by illustrative participant quotes.

### Analytical procedure: pathway mapping

Alongside thematic analysis, participants’ accounts were mapped into a sequential care pathway. In NVivo, process codes such as referral triggers, triage criteria, handovers and discharge protocols were identified and visually mapped to illustrate the patient journey. The draft map was repeatedly checked against transcripts to ensure all key operational steps were included. The draft pathway and themes were then shared with two healthcare professionals and a service lead for feedback. This step, in keeping with RTA, aimed to confirm the pathway’s practical relevance rather than achieve consensus or objective verification. Their comments led to minor adjustments in wording and order, making the final pathway diagram contextually accurate and useful for practice.

### Reflexivity

Reflexivity was enacted continuously throughout the study,^26 27^ with the lead researcher keeping a journal to critically assess how their perspective influenced the analysis. As external members of the Croydon teams, the researchers helped reduce social desirability bias, encouraging candid accounts of systemic issues. Nevertheless, their outsider status meant limited knowledge of local governance. To address this, coding specifically delved into the underlying reasons for operational frustrations, rather than accepting process descriptions at face value. The operationalisation of reflexivity directly influenced theme development. During the initial analytical phases, our positionality as external researchers led us to provisionally code secondary care’s rapid discharge practices as ‘specialist rejection’ or ‘hospital pushback’. However, through continuous reflexive journaling and peer debriefing, this assumption was critically interrogated. We recognised that framing these operational actions as ‘rejection’ assigned undue interpersonal blame to clinicians and obscured the rigid system-level commissioning constraints dictating secondary care capacity. This reflexive pivot fundamentally altered the thematic architecture of the study; it shifted the analytical narrative away from individual clinical reluctance, culminating directly in the systemic theme: ‘The General Practitioner as linchpin in fragmented referral pathways’. This process ensured that the interpretation transcended descriptive interpersonal observations to capture deeper structural realities.

### Patient and public involvement

Patients and/or the public were not directly involved in the design, conduct, reporting or dissemination plans of this research. The study focused exclusively on the operational experiences of healthcare professionals.

## Results

12 NHS healthcare professionals participated in this study. The sample comprised GPs (n=5), respiratory consultants (n=2), specialist nurses (n=3), a respiratory physiotherapist (n=1) and a psychologist (n=1). The participants represented a highly experienced clinical cohort, with professional experience ranging from 3 to 33 years (median=18 years). Of the 12 participants, eight were female and four were male (online supplemental file 3).

Four main themes emerged from a reflexive thematic analysis of the operational reality of the long covid care pathway: navigating diagnostic uncertainty and subjective severity classification; the GP as linchpin in fragmented referral pathways; therapeutic pragmatism and symptom-led support; and the continuity gap and variable discharge practices. An overview of these themes, key concepts and foundational quotes is provided in online supplemental file 4.

### Theme: navigating diagnostic uncertainty and subjective severity classification

Participants described long covid as a highly heterogeneous multisystem condition that created significant diagnostic challenges. Across all interviews, fatigue, breathlessness and cognitive impairment (brain fog) were identified as the hallmark presentations (table 1). The pervasive nature of these core symptoms was noted as the primary driver for patients seeking medical attention:

Fatigue, breathlessness and brain fog are the symptoms I see most frequently (Participant #1, GP).

**Table 1:** Commonly reported long covid symptoms mapped to local specialist referral routes

Symptom cluster	Primary symptoms (reported by >75% of participants)	Secondary symptoms (reported by <75% of participants)	Primary referral route/MDT destination
Respiratory/cardiopulmonary	Breathlessness (dyspnoea)	Chest pain, palpitations, chronic cough	Hospital respiratory clinic/cardiology services
Functional/musculoskeletal	Excessive fatigue	Joint aches (arthralgia), generalised weakness, malaise	Community rehabilitation services/physiotherapy
Cognitive/psychological	Cognitive impairment (brain fog)	Memory loss, mood fluctuations (anxiety/depression)	Psychological services, Improving Access to Psychological Therapies (IAPT)
Systemic/other	—	Sleep disturbances, menorrhagia, loss of smell (anosmia)	GP-led management/specialist nursing

Symptom clusters reflect the commonly reported clinical presentations that guide referral decisions within local long covid care pathways.

GP, general practitioner; MDT, multidisciplinary team.

This multisystem presentation was echoed across secondary and allied health services, where practitioners noted that physical and neurological symptoms frequently co-occurred and significantly impacted baseline function:

Fatigue, brain fog, shortness of breath, joint aches and palpitations are common symptoms we see in rehabilitation (Participant #8, Respiratory Physiotherapist).

Five participants noted that the severity of initial COVID-19 did not predict long-term outcomes, with even mild cases sometimes leading to severe long covid. No formal severity scale was used; instead, 9 of 12 participants described severity classification as subjective, based on clinical judgement and its impact on daily living:

Impact on life, for example, reduced abilities, helps determine the severity of the condition (Participant #2, GP).

This reliance on subjective impact rather than clinical staging was further highlighted by primary care providers as the primary driver for management:

It is subjective, with no classification. The management option is based on the symptoms of the patient and… its impact on the quality of life is most severe (Participant #10, GP).

This lack of formal grading was echoed by allied health professionals, who noted that therapeutic treatment proceeded regardless of formal severity classification:

We just treated them regardless of their severity… in terms of long covid itself, we didn’t use anything, and I didn’t know that there was anything that existed in terms of grading (Participant #8, Respiratory Physiotherapist).

Due to the absence of formal grading, diagnosis was pragmatic and based on exclusion. Ten participants reported that diagnosis depended on taking a thorough history of symptoms persisting over 12 weeks, while systematically ruling out other causes:

Diagnosis is mostly based on a good history and excluding other causes… Persistent symptoms after COVID for more than three months without improvement would suggest long covid (Participant #4, GP).

This exclusionary approach was necessitated by the profound clinical uncertainty surrounding the condition. GPs frequently noted how difficult it was to confidently diagnose the condition given its similarity to other post-viral syndromes, chronic fatigue or health anxiety:

There is no clear diagnostic test or reliable criteria. It can be challenging as there is overlap with a range of other conditions—for instance, chronic fatigue syndromes and health anxiety (Participant #1, GP).

Secondary care practitioners similarly highlighted this ambiguity:

There’s no specific diagnostic test, and the pathophysiology is still poorly understood (Participant #6, Respiratory Consultant).

To navigate this uncertainty and ensure clinical safety before referral, distinct approaches were adopted across professions. While respiratory consultants focused heavily on targeted clinical investigations to rule out specific cardiopulmonary diseases, GPs routinely relied on holistic symptom tracking alongside baseline diagnostic investigations:

We usually arrange blood tests, ECGs, and chest X-rays to rule out other causes (Participant #10, GP).

To support safe referrals without a clear diagnostic biomarker, services adopted structured assessment tools. Several primary care practitioners used digital templates, such as Arden’s (a widely used clinical decision support system in UK primary care),^28^ while the rehabilitation team used screening questionnaires to quantify symptoms:

When we received a referral, we sent a questionnaire, such as the Yorkshire rehabilitation screening tool, to assess symptom severity (Participant #8, Respiratory Physiotherapist).

### Theme: the GP as linchpin in fragmented referral pathways

Participants consistently identified GPs as the central coordinating point within the long covid care pathway. All 12 reported that patients with ongoing long covid symptoms usually first saw their GP, making GPs both initial assessors and administrative gatekeepers responsible for managing uncertainty, coordinating tests and guiding patients to the right services.

Primary care healthcare professionals noted that many cases could be managed in general practice through holistic assessment and support, but complex cases or those needing specialist care required referral:

The majority are manageable in general practice: taking a clear history, performing an examination, formulating a diagnosis, and a management plan are all within my remit. I refer when further investigation or advice/help—beyond my scope—is required (Participant #1, GP).

However, analysis highlighted a major challenge: without an integrated long covid service, healthcare professionals had to split patients’ multisystem symptoms by referring them to organ-specific specialists based on their main symptom:

The majority are manageable in general practice… but in the absence of a clear, commissioned holistic long covid service, referral might be to respiratory, neurological or cardiovascular teams depending on the clinical picture (Participant #1, GP).

Care pathways were determined by patients’ presenting symptoms. Most healthcare professionals sent those with respiratory issues to hospital clinics, while fatigue or reduced function led to direct referral for community rehabilitation, and cognitive or mood problems were routed to psychological services.

Specialist services depended on GPs to triage and refer patients, rather than accepting self-referrals:

I think the best people to speak to would be GPs… because we’d get the majority of our referrals from them (Participant #7, Psychologist).

When we got a referral, we would send them a questionnaire, the Yorkshire Rehabilitation Screening… If they met the criteria, they would then have an assessment with the clinician and the multidisciplinary team (Participant #8, Respiratory Physiotherapist).

Although patients eventually accessed specialist care, the pathway was fragmented, often requiring multiple referrals across separate services. This increased administrative workload in primary care and made navigating the system more complicated for patients.

Healthcare professionals also noted the circular nature of care, with patients frequently discharged back to the GP to coordinate further treatment due to the narrow focus of secondary services:

The patients would be discharged from a multidisciplinary approach, and then referred back to the GP (Participant #8, Respiratory Physiotherapist).

Primary care participants explicitly expressed frustration in reconciling national clinical guidelines with local operational realities. As one GP noted, the theoretical ideal of an integrated clinic often clashed with the fragmented services actually available:

NICE guidance talks about integrated holistic clinics… but in the absence of a clear, commissioned holistic long covid service, a referral might be made to respiratory, neurological, or cardiovascular teams (amongst others), depending on the clinical picture. (Participant #1, General Practitioner).

### Theme: therapeutic pragmatism and symptom-led support

All 12 participants stated that, without disease-modifying treatments for long covid, management was pragmatic and focused on symptoms. Care plans were developed with patients to relieve their most troublesome symptoms, improve function and encourage self-management. Healthcare professionals treated this as an ongoing process, adapting plans based on patients’ responses and tolerances.

A senior primary care healthcare professional highlighted the collaborative and flexible approach to decision-making:

Determining the most appropriate option is through discussion and negotiation with the patient. Options include pulmonary rehab, analgesia, antidepressants/antianxiety medications, physiotherapy, or counselling… which depend on the symptomatology and the patient (Participant #1, GP).

Rehabilitation interventions formed the core of care for patients with fatigue, deconditioning or breathlessness. Healthcare professionals used pacing, graded activity, and condition-specific therapies like breathing retraining, delivered by allied health professionals within a segmented MDT:

If it were physiotherapy, it would be a home exercise programme, or they would join the exercise classes… If it were occupational therapy, it would be an education on fatigue management… If it were psychology, it would be for the brain fog (Participant #8, Respiratory Physiotherapist).

Psychological and peer-support approaches were vital for managing cognitive and emotional symptoms. Practitioners used brief cognitive strategies, low-intensity cognitive behavioural therapy and peer support groups to address difficulties with concentration, low mood and adjustment to ongoing symptoms:

We might be thinking about low-level CBT… if someone has difficulties with cognition, we’d be thinking more about giving them coping strategies and helping them manage their cognition (Participant #7, Psychologist).

Medications were used solely for symptom relief, with GPs prescribing analgesics, inhalers, or short courses of steroids and bronchodilators when needed.

**Figure 1:**
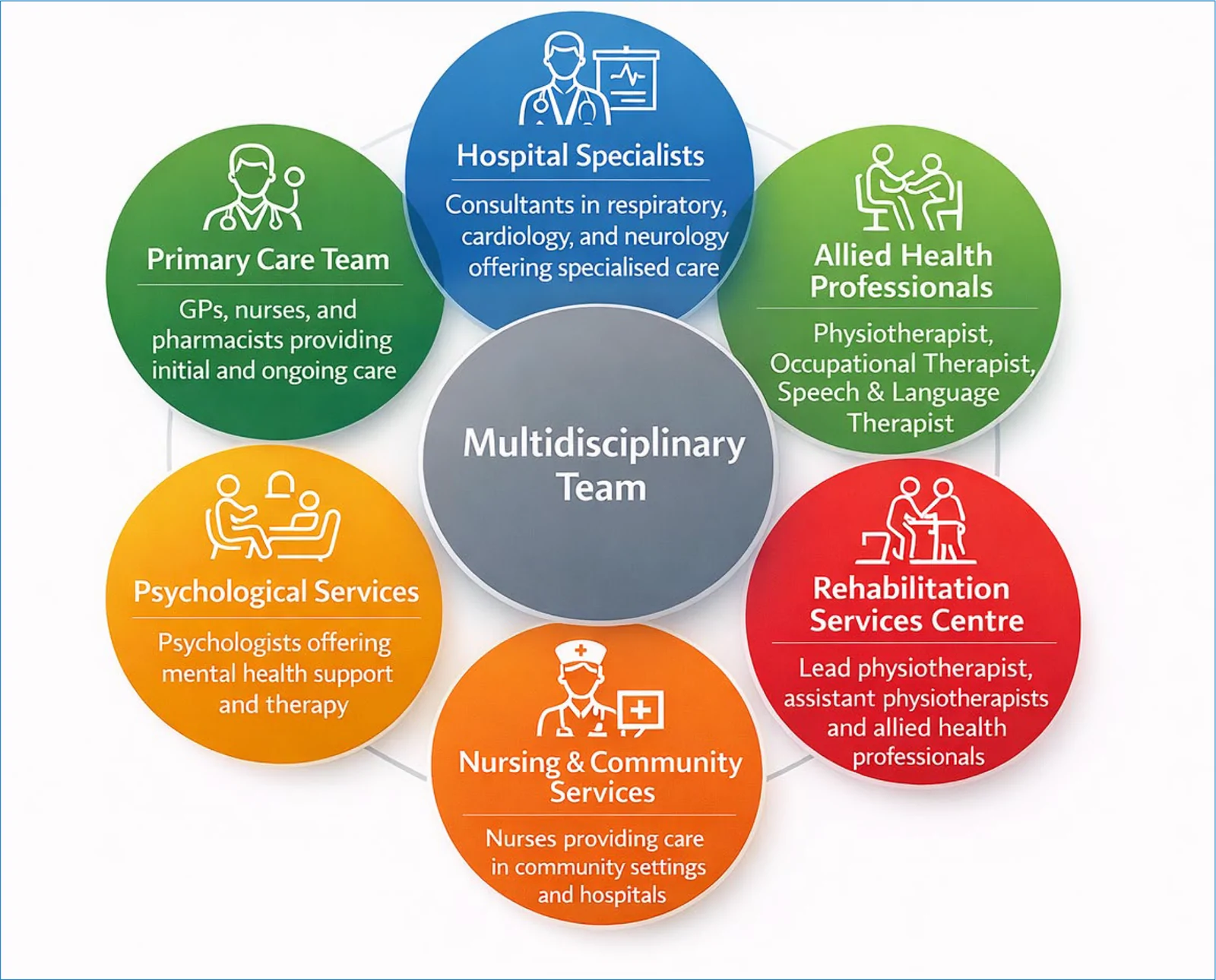
Multidisciplinary team (MDT) composition and interactions in long covid management. This figure shows how primary care, hospital specialists and allied health professionals interact in patient care, based on interviews with 12 NHS practitioners. Overlapping areas mark collaborative care; separate ones highlight distinct specialist referrals. GP, general practitioner; NHS, National Health Service.

Delivering symptom-led pharmacological and rehabilitative care required a broad MDT. Figure 1 illustrates the MDT’s composition and the interfaces between primary care, hospital specialists and allied health professionals. Although all participants stressed the importance of MDT collaboration, four described inconsistent execution. Where regular case conferences involving the GP and the broader MDT, and unified records, existed, coordination was seamless; elsewhere, it relied on ad hoc emails and informal negotiations, increasing practitioners’ administrative workload.

At the long covid rehabilitation centre, we collaborated closely with occupational therapists, physiotherapists, psychologists, psychiatrists, and the speech-language therapist. We communicated constantly, since we were in the same building. We have dedicated times for meetings and communication to address patient care and management (Participant #8, Respiratory Physiotherapist).

### Theme: the continuity gap and variable discharge practices

Continuity of care after specialist assessment was a recurring issue. Eight of twelve participants said long-term follow-up mainly relied on patients contacting their GP, rather than regular, scheduled monitoring by secondary or rehab services. GPs noted that ongoing care often depended on patients returning if symptoms persisted or worsened:

We often rely on patient-initiated contact. I can review long covid symptoms when assessing other conditions (Participant #5, GP).

Patients will generally return to the same GP to report in and discuss progress (or lack of progress) (Participant #1, GP).

Discharge decisions from rehabilitation were made jointly by the MDT, based on patients reaching functional goals or when further improvement stopped. Healthcare professionals used standard outcome measures and set programme durations before discharge:

Patients stay 6–12 months in the rehab before being discharged. We use some outcome measures… we reassess them at the end and see if they have improved in their symptoms (Participant #8, Respiratory Physiotherapist).

Despite formal measurement, post-discharge monitoring varied greatly among participants. Rehabilitation teams stopped tracking patients after sending discharge summaries to primary care:

No, we do not follow up with the patient we send to the GP and discharge to the GP, and we do not follow up again (Participant #8, Respiratory Physiotherapist).

Consequently, patients had to reach out to their GP for follow-up, creating a continuity gap, particularly challenging for those with fluctuating symptoms or limited ability to self-advocate.

Without long-term specialist tracking, healthcare professionals mainly relied on subjective and functional outcomes—such as returning to work or baseline activities—to gauge recovery:

Patient progress is known when presenting signs and symptoms are reduced or no longer present (Participant #11, Specialist Nurse).

### Care pathway mapping

**Figure 2:**
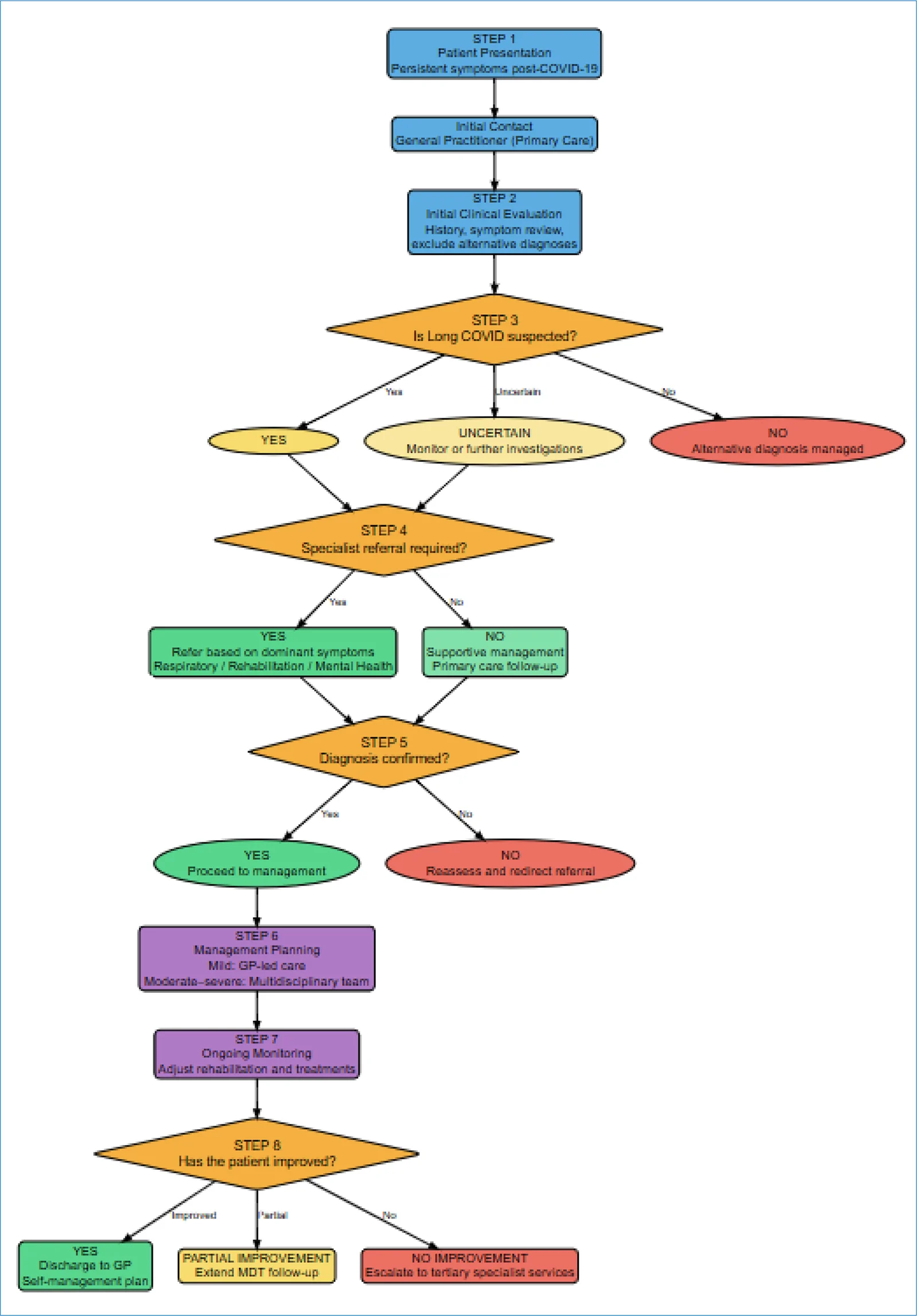
Sequential care pathway for the management of long covid. This flowchart maps the ‘lived’ trajectory of an adult patient from initial primary care presentation to eventual discharge, derived from the thematic synthesis of healthcare professional interviews. Diamond nodes represent critical clinical decision points (eg, triage and diagnostic confirmation), while rectangular nodes represent operational management actions. Decision nodes (eg, ‘Confirmed as Long covid?’) represent administrative labels required to navigate clinical triage and unlock service funding, rather than absolute biological certainty. GP, general practitioner; MDT, multidisciplinary team.

Thematic analysis of participants’ experiences led to an eight-step CCP, summarised below and mapped in figure 2 to show a patient’s journey from first contact to discharge, highlighting key decision points. It is important to note that a ‘Yes’ or ‘Confirmed’ decision within this pathway does not represent biological certainty. Rather, it represents an administrative or pragmatic label that clinicians are compelled to apply to a patient to unlock funding or trigger a referral to a specialist clinic, highlighting the core tension between bureaucratic decision trees and underlying clinical ambiguity. The analytic audit trail demonstrated the progression from raw data to a mapped pathway (online supplemental file 5):

- Step 1: Patient presents with ongoing long covid symptoms, usually to a GP.
- Step 2: GP assesses symptoms, excludes other causes, and acts as gatekeeper for referrals.
- Step 3: If long covid is suspected, the pathway continues; if unclear, the patient is monitored or referred for further tests.
- Step 4: Referral is made depending on symptoms—respiratory clinic, rehabilitation or psychological services; otherwise, GP provides support and self-management advice.
- Step 5: Diagnosis is confirmed clinically by GP or specialist, after ruling out other causes.
- Step 6: MDT assesses impact. Mild cases are managed by a GP; moderate/severe cases involve MDT care with a coordinator.
- Step 7: Ongoing reviews and treatment adjustments are provided as needed.
- Step 8: Discharge to GP with summary if stable; MDT follow-up or escalation if not improved.

## Discussion

This study mapped the operational reality of the long covid care pathway within a diverse NHS setting. The central interpretive insight is that frontline management of long covid is currently defined by the pragmatic navigation of clinical uncertainty.^29^ While national guidelines provide theoretical frameworks, healthcare professionals in this study reported significant challenges in translating these into cohesive practice. Consistent with national health reports, participants confirmed that patients who experienced only mild acute COVID-19 infections frequently developed severe, long-term complications.^30^ However, the absence of definitive biomarkers necessitates a heavy reliance on subjective assessments and exclusionary diagnostic pathways.

The findings reveal a stark contrast between literature and frontline reality regarding severity classification. While various outcome measures have been developed, such as the post-COVID Functional Status scale,^31^ these were rarely used by participants. Instead, severity was determined subjectively based on the functional impact on daily living. To navigate diagnostic uncertainty, practitioners employed a pragmatic, exclusionary approach, using routine blood tests and basic imaging to rule out alternative pathologies. To bridge this gap and standardise the influx of referrals, local services implemented structured tools, such as the Ardens long covid template^28^ and the Yorkshire rehabilitation screening tool.^32 33^ Ardens is a comprehensive clinical decision support system embedded within primary care electronic health records across the NHS.^34^ The rapid deployment of the Ardens long covid template allowed GPs to operationalise the NICE guidelines at the point of care.^2^ By providing a structured interface for comprehensive history-taking, prompting necessary exclusionary investigations (eg, chest radiographs and specific blood panels), and embedding validated screening tools (eg, the Patient Health Questionnaire-2 for depression), the template scaffolds the clinical assessment process.^34^ The reliance on local templates underscores a system self-organising to manage risk in the absence of a single, nationally mandated diagnostic test.

Moreso, referral and triage practices varied significantly, largely driven by local service capacity rather than clinical ideals. National guidelines, such as those from NG188, explicitly call for integrated referral pathways linking primary care with multidisciplinary rehabilitation and specialist clinics.^2^ However, this study found that true integration remains aspirational. In practice, GPs functioned as the administrative linchpins, required to triage patients into fragmented, symptom-specific streams. Rather than accessing a single hub, patients with respiratory symptoms were directed to hospital respiratory clinics, while those with fatigue bypassed medical clinics and went directly to community rehabilitation. This siloing contradicts the multisystem nature of the disease and places an immense coordinating burden on primary care.

Participants’ data from this study are congruent with current literature; management is predominantly focused on symptom relief, rehabilitation and self-management.^35 36^ No curative pharmacotherapy currently exists for long covid.^36^ Consequently, participants described a treatment paradigm heavily reliant on non-pharmacological interventions. Examples included energy-conservation strategies (pacing), cognitive strategies for ‘brain fog’, and, specifically, breathing retraining to manage dysfunctional breathing patterns, aligning with recent consensus guidelines from the Association of Chartered Physiotherapists in Respiratory Care.^37^

Pharmacological interventions were used sparingly and strictly for symptomatic relief (eg, analgesics for arthralgia or inhalers for airway hyperreactivity), contradicting the notion of a heavily medicalised pathway. It is important to contextualise that at the time of data collection (mid-to-late 2024), definitive, disease-modifying pharmacotherapy was not yet widely established in routine NHS practice. While the evidence base is rapidly evolving—with recent clinical trials demonstrating promising targeted pharmacological interventions for specific symptoms such as fatigue, ^38^these novel options currently address isolated outcomes rather than providing a holistic cure for the condition’s complex, multisystem presentation. Therefore, the reliance on a rehabilitation-centred approach coordinated by MDT, and the careful monitoring of functional outcomes, remains operationally valid and closely mirrors national guidelines.^36^ However, as emerging pharmacological therapeutics mature and formally enter clinical commissioning pathways, the operational reality mapped in this study must remain dynamic. The introduction of definitive medical treatments will likely fundamentally alter the future GP-specialist dynamic, necessitating iterative updates to the care pathway.

Continuity of care emerged as a critical systemic vulnerability. The literature demonstrates that ongoing, patient-centred care is vital for managing fluctuating conditions; a recent UK-based scoping review of primary care concluded that consistent relationships improve clinical management in long covid.^39^ However, our findings identified a ‘continuity gap’ post-discharge. While some practices supported continuity through unified digital records or case conferences (involving the GP and the MDT), many secondary services discharged patients without scheduled follow-up. This placed the burden of re-initiating care entirely on the patient. Addressing this gap is critical, as patient-facing studies indicate that healthcare professionals’ empathy, validation, and sustained continuity significantly impact patients’ perceived quality of care.^40^ The healthcare professionals in this study echoed global health policy discussions, confirming that capacity shortfalls and service fragmentation are major barriers to delivering the ‘one-stop’ care models advocated by the WHO and NHS England.^2 40^ This continuity gap intersects critically with local health inequalities. In a demographically diverse and socioeconomically mixed area like Croydon, a pathway reliant on patient-initiated follow-up disproportionately disadvantages individuals with lower health literacy, language barriers or limited capacity to aggressively self-advocate for re-referral. Therefore, the lack of proactive post-discharge monitoring not only creates an administrative gap but risks widening post-COVID health disparities.

Our pathway differs significantly from previous NHS care models. For instance, the patient pathway mapped by the STIMULATE-ICP study, based on data collected between November 2022 and July 2023, revealed early, successful efforts to establish highly integrated, dedicated long covid clinics.^15 16^ By contrast, our 2024 data show a systemic regression towards fragmented, symptom-specific referrals. As dedicated pandemic-era commissioning has evolved and capacity constraints have tightened, symptoms such as breathlessness are increasingly routed back to standard respiratory clinics rather than to dedicated multidisciplinary hubs. This highlights the vulnerability of novel care models when ring-fenced funding ceases, demonstrating a shift from integrated innovation back to traditional siloed care. For policymakers and commissioners, it is essential to distinguish between challenges that are intrinsic to the disease biology and those that are remediable through service design. Intrinsic biological barriers, such as the lack of a definitive biomarker and multisystem heterogeneity, drive diagnostic uncertainty, which cannot be immediately resolved by health policy. In contrast, remediable service design barriers, such as siloed single-organ referral systems, the lack of funded MDT case conferences and poor post-discharge digital tracking, are entirely within the control of local commissioners to optimise.

### Limitations and future directions

The limitations of this study must be acknowledged. First, the sample size (n=12), while yielding sufficient ‘information power’ for the highly specific aim of mapping a local pathway, was drawn exclusively from a single, diverse borough in South London (Croydon). Because service commissioning, funding mechanisms and operational pathways vary significantly across different NHS Trusts and international contexts, the exact referral routes mapped here cannot be universally applied. In qualitative research, however, the aim is not statistical generalisability but rather conceptual transferability. The underlying systemic frictions identified—such as the administrative ‘gatekeeper burden’ placed on primary care and the challenges of operationalising subjective severity without definitive biomarkers—resonate strongly with broader health services literature. As health systems transition from acute pandemic responses to managing complex chronic sequelae, traditional siloed structures are frequently challenged. To aid policymakers in navigating similar resource-constrained environments, online supplemental file 6 synthesises these themes into conceptually transferable insights, mapping them against potential service design levers (eg, closing the ‘continuity gap’ through formal post-discharge tracking). Second, the self-selection of participants could introduce recruitment bias.^41^ As established in qualitative methodological literature, healthcare professionals who actively volunteer for interview studies often possess stronger opinions, specific frustrations or heightened interest regarding pathway inefficiencies compared with those who opt not to participate. Third, the study design relied on asynchronous email interviews for a minority of participants (n=3). While a formal statistical sensitivity analysis is not applicable to Reflexive Thematic Analysis, a reflexive comparison of the datasets revealed distinct modality differences. Email respondents provided highly structured, measured and policy-focused answers (likely due to the ability to edit their text), whereas synchronous interviews yielded more spontaneous expressions of emotional burnout and operational frustration. We acknowledge that this modality difference influenced the emotional depth of the data captured from those specific participants. Finally, because this study focused exclusively on the provider perspective, the mapped flowchart represents the provider-perceived operational pathway, which inherently lacks the lived reality of the patients navigating it. Moving forward, synthesising these provider and patient perspectives will be vital. As highlighted in recent literature, any future pathway redesign must use patient co-design and participatory methods; integrating patient-reported experiences, priorities and contextual factors into service development is essential for improving the relevance, acceptability and overall effectiveness of novel care models.^42^

## Conclusions

As healthcare systems shift from acute COVID-19 response to chronic care, long covid presents significant operational challenges. This study, involving 12 NHS healthcare professionals, found management is shaped by diagnostic uncertainty and fragmented services. Without clear biomarkers, diagnosis depends on subjective symptom reporting and clinical judgement and the exclusion of alternative conditions. Management focuses mainly on symptom-led rehabilitation, favouring non-pharmacological approaches (like pacing and breathing retraining), with limited, targeted pharmacological treatments for short-term relief. Referral patterns centred on GP-led initiation and coordination, with patients triaged to respiratory, rehabilitation or mental health services based on symptom profiles. Fragmented pathways and inconsistent discharge practices often leave patients facing a post-discharge continuity gap. The study emphasises the need to standardise assessment tools, diagnostic criteria and referral processes. National guidelines set the ideal, but bridging the gap locally is crucial. Rebuilding services into integrated, person-centred care pathways should be a structural priority to improve patient outcomes and sustain the healthcare workforce.

## Supplementary material

10.1136/bmjopen-2026-120704 online supplemental file 1

## Data Availability

Data are available upon reasonable request.
