# Supplementary material for: Exploring long covid care pathways in the UK NHS: a qualitative study of healthcare professionals’ experiences
Source: BMJ Open. 2026 Sep 15;16(9):e120704. doi: 10.1136/bmjopen-2026-120704 (PMC13583841; doi:10.1136/bmjopen-2026-120704)
Supplement: Supplementary file 1 [file bmjopen-2026-120704.reviewer_comments.pdf]

PEER REVIEW HISTORY

BMJ Open publishes all reviews undertaken for accepted manuscripts. Reviewers are asked to complete a checklist review form and are provided with free text boxes to elaborate on their assessment. These free text comments are reproduced below.

ARTICLE DETAILS

Title (Provisional)

Exploring Long COVID Care Pathways in the UK NHS: A Qualitative Study of Healthcare Professionals' Experiences

Authors

Daodu, Lanre; RASTE, Yogini; Allgrove, Judith E.; Arrigoni, Francesca; Kayyali, Reem

---

VERSION 1 - REVIEW

**Reviewer**	**1**
**Name**	**Ramasawmy, Mel**
**Affiliation**	**Queen Mary University of London, Institute of Health Informatics**
**Date**	**11-May-2026**
**COI**	**None**

Overall this is a clearly presented study which describes experiences of implementing Long Covid care within a local system. I have included some specific and general comments below:

Introduction

The authors have cited work of both STIMULATE-ICP and LOCOMOTION studies, both of which included qualitative work on HCPs' experiences of Long Covid care pathways. I would like to see a strengthened explanation of what additional or new insights they aimed to explore (e.g. what had changed in Long Covid pathway commissioning by the time this data was collected, structure and challenges of the local system, etc.)

Methods

Overall the methods appear appropriate, and the decision to offer email participation is justified. I note that the authors describe that the median input by email was approx. 800 words. Did the authors feel that this was sufficient detail? (Transcription of a typical 30 minute interview will yield a much higher word count).

Findings

The findings presented are interesting and resonate with previous research. As the aim of the paper was to highlight challenges of translating guidelines into practice, it would be helpful to include these within the findings, and to include any HCP quotes which address this issue directly.

Discussion

How does the 'pragmatic' pathway shown in figure 1 map to NHS models of care at the time? We produced a similar figure of the patient pathway within STIMULATE-ICP (https://doi.org/10.1136/bmjopen-2023-080967, figure 1), based on data collected between November 2022 and July 2023. It would be interesting to compare any changes (if any) based on emerging evidence for models of support between then (or other papers published at the time), and the data collection for this study.

I would have liked to see a more thorough discussion of the strengths and limitations of the study (see methods section).

General comments

Can the authors provide additional details related to the site and service? What are the general challenges (e.g. relating to systems, inequalities) and how do they intersect with the delivery of Long Covid services?

**Reviewer**	**2**
**Name**	**Milic, Jovana**
**Affiliation**	**University of Modena and Reggio Emilia Faculty of Medicine and Surgery**
**Date**	**10-Jun-2026**
**COI**	**None**

This qualitative study explores how NHS healthcare professionals in the London Borough of Croydon operationalise Long COVID care pathways in practice. Using single-timepoint semi-structured interviews with 12 multidisciplinary clinicians, including GPs (n=5), respiratory consultants (n=2), specialist nurses (n=3), a physiotherapist, and a clinical psychologist, data were collected between July and October 2024. Analysis followed Braun and Clarke's Reflexive Thematic Analysis (RTA) framework.

Four central themes emerged: (i) diagnostic uncertainty navigated through exclusionary testing; (ii) GPs functioning as administrative gatekeepers in fragmented, symptom-driven referral systems; (iii) therapeutic pragmatism centred on non-pharmacological rehabilitation; and (iv) a post-discharge "continuity gap" placing re-engagement burden on patients. These were synthesised into an eight-step operational care pathway. The authors conclude that while national guidelines (NICE NG188) set a theoretical standard, local implementation is

hampered by absent biomarkers, siloed specialist services, and inconsistent post-discharge monitoring. Recommendations include standardising functional assessment tools, establishing genuinely integrated interdisciplinary clinics, and introducing robust post-discharge tracking systems. While the research addresses an important healthcare challenge, there is a notable gap between the study's methodological scope as a single-borough setting with a healthcare-professional-only perspective and its broader aspirations for NHS-wide and international applicability. Addressing this, along with clarifying several unresolved analytical contradictions and substantiating reflexivity claims, would strengthen the paper's credibility and impact for service redesign and implementation science.

MAJOR ISSUES

1. Claims of NHS-wide and international applicability (Discussion, lines 501–509) lack empirical justification for a single-borough study. What evidence supports generalizability to other NHS Trusts with potentially different demographics, funding, and service structures?

2. The study maps the "lived operational pathway" while excluding patients themselves. How does excluding the central actors navigating this pathway support claims of operational validity? Patients' unmet needs should be considered when creating a pathway support. Approaches based on co-design and participatory methods have been shown to improve the relevance, acceptability, and effectiveness of care models by integrating patient-reported experiences, priorities, and contextual factors into service development (see Zhuang et al 2025 https://doi.org/10.1016/j.diabres.2025.112028 or Attal et al 2025 https://doi.org/10.1186/s12913-025-12423-3).

3. Supplementary File S3 shows P7 (Psychologist) has 3 years and P8 (Physiotherapist) 5 years of clinical experience. Did both meet the six-month long COVID-specific experience criterion? How years of experience have affected response?

4. Claims of rigorous reflexivity lack concrete evidence. Provide specific examples of how the researchers' positionality actually altered coding, theme construction, or interpretation, not just procedural descriptions.

5. 25% of data (n=3) came from asynchronous email interviews (~800 words each), 75% from synchronous audio-recorded interviews (25–45 minutes). These interactional modalities produce fundamentally different data. Were sensitivity analyses conducted comparing themes by modality? Could email respondents have self-selected for different disclosure patterns?

6. The paper dismisses pharmacological treatment (line 473: "no curative pharmacotherapy") yet was conducted July–October 2024 with April 2026 submission. Does the pathway remain valid as pharmacological options and evidence emerge (e.g. https://www.acpjournals.org/doi/10.7326/ANNALS-25-03959).

7. The flowchart presents "Is Long COVID suspected?" as a binary YES/UNCERTAIN/NO decision despite the preceding theme emphasizing profound diagnostic uncertainty. Does

the visualization adequately represent operational reality, or does it suggest deterministic decision-making?

8. The paper identifies systemic challenges but does not distinguish between barriers intrinsic to disease biology (lack of biomarkers) versus remediable through service design (lack of integration). For policymakers, this distinction is essential.

VERSION 1 - AUTHOR RESPONSE

Response to Reviewer 1

Exploring Long COVID Care Pathways in the UK NHS: A Qualitative Study of Healthcare Professionals' Experiences

Submission ID: bmjopen-2026-120704

We would like to express our sincere gratitude for the rigorous, constructive, and highly insightful feedback provided on our manuscript. We have carefully considered all comments, which have fundamentally strengthened the methodological transparency, analytical depth, and international relevance of our work. Below, we provide a detailed, point-by-point response outlining exactly how we have addressed each concern and incorporated your recommendations into the revised manuscript.

	Reviewer comment	Author responses
	**Introduction**	
1	The authors have cited work of both STIMULATE-ICP and LOCOMOTION studies, both of which included qualitative work on HCPs' experiences of Long Covid care pathways. I would like to see a strengthened explanation of what additional or new insights they aimed to explore (e.g. what had changed in Long Covid pathway commissioning by the time this data was collected, structure and challenges of the local system, etc.)	**Response 1:** Thank you for highlighting this. We have revised the Introduction to clearly define our temporal and contextual gap. We now explicitly state that while STIMULATE-ICP and LOCOMOTION mapped pathways during periods of dedicated, ring-fenced national funding (2021–2023), our data (mid-to-late 2024) captures a transition phase. We highlight that as dedicated funding mechanisms shifted or ceased, the operational burden heavily pivoted back to routine primary care within a highly diverse, resource-constrained borough.<br><br>Revision :<br>"Significant progress has been made in defining best-practice models for long COVID care, notably through large-scale initiatives such as the NIHR-funded LOCOMOTION and STIMULATE-ICP studies, which established 'gold standard' pathways for assessment and

		rehabilitation. 15 16. However, these foundational studies mapped care pathways between 2021 and 2023, during a period of dedicated, ring-fenced national funding for long COVID services. As these initial funding mechanisms have shifted or ceased, the operational burden has pivoted heavily back to routine primary care. Our data, collected in mid-to-late 2024, captures this critical transition phase. Translating high-level protocols into operational reality remains a significant challenge for individual NHS Trusts, particularly those adapting to the loss of dedicated pandemic-era resources. …….
	**Methods**	
2	Overall the methods appear appropriate, and the decision to offer email participation is justified. I note that the authors describe that the median input by email was approx. 800 words. Did the authors feel that this was sufficient detail? (Transcription of a typical 30 minute interview will yield a much higher word count).	**Response 2:** This is a very valid methodological point. While the absolute word count of an email exchange is lower than a spoken transcript, we found the data density (Information Power) to be sufficient because participants had time to meticulously reflect on and refine their written answers. However, we agree this modality difference must be acknowledged. We have added a paragraph to the Limitations section explicitly noting that, while email enabled us to reach severely time-poor senior clinicians, it lacked the spontaneity and volume of synchronous interviews.<br><br>Revision :<br>Methods section<br>"These multi-stage exchanges yielded a median of approximately 800 words per participant. While the absolute word count of an asynchronous email exchange is naturally lower than a spoken transcript, the data density (Information Power) was judged to be high, as participants had the opportunity to meticulously reflect on and refine their written answers."[25]<br><br>Strengths and Limitations section<br>Add<br>• The study design relied on asynchronous email interviews for

		a minority of participants (n=3). While this modality enabled the inclusion of severely time-poor senior clinicians and yielded highly structured, reflective responses, it inherently lacked the spontaneity, emotional depth, and raw volume of data generated by synchronous audio-recorded interviews.
	**Findings**	
3	The findings presented are interesting and resonate with previous research. As the aim of the paper was to highlight challenges of translating guidelines into practice, it would be helpful to include these within the findings, and to include any HCP quotes which address this issue directly.	**Response 3:** We have updated the Results section to include specific quotes where participants express frustration with guidelines versus reality. For example, under Theme 2, we added a quote highlighting the tension between the NICE recommendation for "integrated holistic clinics" and the reality of the local system, which forced GPs to send patients to fragmented, single-organ specialities.<br><br>Revision :<br>Primary care participants explicitly expressed frustration when trying to reconcile national clinical guidelines with local operational realities. As one GP noted, the theoretical ideal of an integrated clinic often clashed with the fragmented services actually available:<br><br>*'NICE guidance talks about integrated holistic clinics... but in the absence of a clear, commissioned holistic long COVID service, referral might be made to respiratory, neurological or cardiovascular teams (amongst others) depending on the clinical picture.' (Participant 1, General Practitioner)"*
	**Discussion**	
4	Discussion<br>How does the 'pragmatic' pathway shown in figure 1 map to NHS models of care at the time? We produced a similar figure of the patient pathway within STIMULATE-ICP (https://doi.org/10.1136/bmjopen-2023-	**Response 4**: Thank you for directing us to this excellent paper. We have added a dedicated paragraph to the Discussion comparing our mapped pathway (late 2024) to the STIMULATE-ICP pathway (2022/2023). We highlight a notable shift:

	080967, figure 1), based on data collected between November 2022 and July 2023. It would be interesting to compare any changes (if any) based on emerging evidence for models of support between then (or other papers published at the time), and the data collection for this study.<br><br>I would have liked to see a more thorough discussion of the strengths and limitations of the study (see methods section).	whereas the STIMULATE-ICP pathway demonstrated early efforts at highly integrated, dedicated Long COVID clinics, our 2024 data reveals a systemic regression toward fragmented, symptom-specific referrals (e.g., routing breathlessness to standard respiratory clinics rather than a dedicated MDT) due to capacity constraints and evolving commissioning landscapes. We have cited the STIMULATE-ICP paper to support this longitudinal comparison.<br><br>Revision:<br>Our mapped pathway presents a striking contrast to earlier NHS models of care. For instance, the patient pathway mapped by the STIMULATE-ICP study—based on data collected between November 2022 and July 2023—demonstrated early, successful efforts to establish highly integrated, dedicated Long COVID clinics.[15 16] In contrast, our 2024 data reveals a systemic regression toward fragmented, symptom-specific referrals. As dedicated pandemic-era commissioning has evolved and capacity constraints have tightened, symptoms such as breathlessness are increasingly routed back to standard respiratory clinics rather than dedicated multidisciplinary hubs. This highlights the vulnerability of novel care models when ring-fenced funding ceases, demonstrating a shift from integrated innovation back to traditional siloed care.<br><br>As noted in our response to the Editor, the Strengths and Limitations section has been completely rewritten and expanded to rigorously address sampling, modality, and epistemological constraints.
	**General comments**	
5	Can the authors provide additional details related to the site and service? What are the general challenges (e.g. relating to systems, inequalities) and how do they intersect with the delivery of Long Covid services?	**Response 5**: We have expanded the "Study Design and Setting" section in the Methods. We now provide context on the London Borough of Croydon, noting its high demographic diversity and pockets

		of high socioeconomic deprivation. In the Discussion (under "The Continuity Gap"), we explicitly link this demographic context to our findings, noting that a pathway reliant on patient-initiated follow-up disproportionately disadvantages patients with lower health literacy or those facing socioeconomic inequalities.<br><br>Revision:<br>This study employed a qualitative design utilising single-timepoint, semi-structured interviews and was conducted within the NHS in the London Borough of Croydon. The London Borough of Croydon was selected as it is a densely populated and highly diverse urban area, characterised by significant variations in socioeconomic status and pockets of high deprivation, making it an ideal setting to explore the intersection of resource constraints and post-pandemic recovery.

Response to Reviewer 2

Exploring Long COVID Care Pathways in the UK NHS: A Qualitative Study of Healthcare Professionals' Experiences

Submission ID: bmjopen-2026-120704

We would like to express our sincere gratitude for the rigorous, constructive, and highly insightful feedback provided on our manuscript. We have carefully considered all comments, which have fundamentally strengthened the methodological transparency, analytical depth, and international relevance of our work. Below, we provide a detailed, point-by-point response outlining exactly how we have addressed each concern and incorporated your recommendations into the revised manuscript.

	Reviewer comments	Authors responses
	**MAJOR ISSUES**	
1	Claims of NHS-wide and international applicability (Discussion, lines 501–509) lack empirical justification for a single-borough study. What evidence supports generalizability to other NHS Trusts with potentially different demographics, funding, and service structures?	**Response 1:**<br>We entirely agree with the reviewer that our previous phrasing overreached and lacked empirical justification for a single-borough study. We have heavily revised this section (previously lines 501–509) to tone down claims of broad 'applicability.' We now

		explicitly acknowledge that differing demographics, funding mechanisms, and service commissioning structures across the NHS and internationally prevent the direct operational generalisation of this pathway. Instead, we have framed our findings strictly through the qualitative lens of conceptual transferability. We clarify that while the exact referral structures are unique to Croydon, the underlying systemic frictions—such as the administrative 'gatekeeper burden' and the challenge of managing chronic disease in siloed structures—represent conceptual insights that may resonate with policymakers navigating similarly resource-constrained environments.<br><br>Revision:<br>Discussion section<br>"Because this study was conducted within a single, demographically diverse London borough, it must be acknowledged that specific operational pathways, funding mechanisms, and service commissioning models vary significantly across different NHS Trusts and international contexts; therefore, the exact referral routes mapped in this study cannot be universally applied. However, the underlying systemic frictions identified—such as the administrative 'gatekeeper burden' placed on primary care and the challenges of operationalising subjective severity without definitive biomarkers—resonate with broader health services literature on chronic disease management. As health systems transition from acute pandemic responses to managing complex chronic sequelae, traditional siloed structures are frequently challenged. Supplementary File S6 synthesises these themes into conceptually transferable insights, mapping them against potential service design levers. For example, while the exact implementation of post-discharge tracking will depend heavily on local demographics and funding structures, the need to close the 'continuity gap' to ensure equitable, person-centred care remains highly

		relevant for policymakers navigating similar resource-constrained environments."
2	The study maps the "lived operational pathway" while excluding patients themselves. How does excluding the central actors navigating this pathway support claims of operational validity? Patients' unmet needs should be considered when creating pathway support. Approaches based on co-design and participatory methods have been shown to improve the relevance, acceptability, and effectiveness of care models by integrating patient-reported experiences, priorities, and contextual factors into service development (see Zhuang et al 2025 https://doi.org/10.1016/j.diabres.2025.112028 or Attal et al 2025 https://doi.org/10.1186/s12913-025-12423-3).	**Response 2:**<br>We sincerely thank the reviewer for this critical observation, and we completely agree that patient-reported experiences are absolutely central to evaluating true operational validity. To clarify the scope of this paper, focuses on one arm of a larger, five-arm comprehensive research programme investigating Long COVID care in Croydon. While this specific manuscript focuses exclusively on the provider-perceived operational pathway and health systems bottlenecks, a separate, dedicated research arm of our wider project focuses entirely on the lived experiences and unmet needs of the patients navigating this system.<br>We have added a paragraph to the 'Strengths and limitations' and 'Future research directions' sections to provide this context. Furthermore, we entirely agree with the reviewer regarding the necessity of participatory methods for subsequent service redesign. We now explicitly state that the ultimate goal is to integrate the findings from this provider arm with the upcoming patient arm to facilitate future co-design. We have incorporated the excellent suggested references (Zhuang et al., 2025; Attal et al., 2025) to highlight how integrating patient priorities improves the relevance and effectiveness of care models.
3	Supplementary File S3 shows P7 (Psychologist) has 3 years and P8 (Physiotherapist) 5 years of clinical experience. Did both meet the six-month long COVID-specific experience criterion? How years of experience have affected response?	**Response 3:**<br>Yes, both met the inclusion criteria, as they were the dedicated clinical leads for the Long COVID psychological and physiotherapy streams, respectively. The years of experience influenced perspectives: senior GPs (20+ years) frequently focused on systemic, administrative, and commissioning frustrations, whereas more junior allied health professionals (3-5 years) focused heavily on protocol adherence, functional outcome measures, and localised triage mechanisms.
4	Claims of rigorous reflexivity lack concrete evidence. Provide specific examples of how the researchers' positionality actually altered coding,	**Response 4**:<br>We thank the reviewer for pushing us to demonstrate reflexivity in action, rather than

	theme construction, or interpretation, not just procedural descriptions.	merely describing it as a procedural step. We completely agree that concrete evidence of how positionality altered the analysis is essential for RTA. We have expanded the 'Reflexivity' section in the Methods to include a specific, transparent example of how our assumptions were challenged and how this fundamentally altered our theme construction.<br>Specifically, we detail how our initial 'outsider' perspective led us to provisionally code strict hospital discharge criteria as 'specialist rejection' or 'hospital pushback'. However, through reflexive journaling and peer debriefing, we actively interrogated this assumption. We realised that framing this as 'rejection' overlooked the rigid commissioning rules that constrained secondary care practitioners. This reflexive pivot shifted our entire analytical narrative away from a theme of 'hospital reluctance' toward our final constructed theme: 'The GP as linchpin in fragmented referral pathways.' We have added this exact example to the manuscript to substantiate our claims of rigorous reflexivity.<br><br>Revision<br>Method section<br>"The operationalisation of reflexivity directly influenced theme development. During the initial analytical phases, our positionality as external researchers led us to provisionally code secondary care's rapid discharge practices as 'specialist rejection' or 'hospital pushback.' However, through continuous reflexive journaling and peer debriefing, this assumption was critically interrogated. We recognised that framing these operational actions as 'rejection' assigned undue interpersonal blame to clinicians and obscured the rigid, system-level commissioning constraints dictating secondary care capacity. This reflexive pivot fundamentally altered the thematic architecture of the study; it shifted the analytical narrative away from individual clinical reluctance, culminating directly in the systemic theme: 'The General Practitioner as linchpin in fragmented referral pathways.'

		Ultimately, this process ensured that the interpretation transcended descriptive, interpersonal observations to capture deeper structural realities."
5	25% of data (n=3) came from asynchronous email interviews (~800 words each), 75% from synchronous audio-recorded interviews (25–45 minutes). These interactional modalities produce fundamentally different data. Were sensitivity analyses conducted comparing themes by modality? Could email respondents have self-selected for different disclosure patterns?	**Response 5**:<br>We have added a discussion of this to our Limitations section. While a formal statistical sensitivity analysis is not applicable to RTA, we reflexively compared the datasets. We note that email respondents provided highly structured, measured, and policy-focused answers (likely due to the ability to edit their text), whereas synchronous interviews yielded more spontaneous expressions of emotional burnout and frustration. We acknowledge that this modality difference may have influenced the emotional depth of the data captured from those specific participants.<br><br>Revision<br>Discussion section<br>….the study design relied on asynchronous email interviews for a minority of participants (n=3). While a formal statistical sensitivity analysis is not applicable to Reflexive Thematic Analysis, a reflexive comparison of the datasets revealed distinct modality differences. Email respondents provided highly structured, measured, and policy-focused answers (likely due to the ability to edit their text), whereas synchronous interviews yielded more spontaneous expressions of emotional burnout and operational frustration. We acknowledge that this modality difference influenced the emotional depth of the data captured from those specific participants.
6	The paper dismisses pharmacological treatment (line 473: "no curative pharmacotherapy") yet was conducted July–October 2024 with April 2026 submission. Does the pathway remain valid as pharmacological options and evidence emerge (e.g. https://www.acpjournals.org/doi/10.7326/ANNALS-25-03959).	**Response 6:** "We thank the reviewer for highlighting the chronological gap between our data collection and submission, and for directing us to the recent Annals of Internal Medicine publication. We agree that the pharmacological landscape is evolving, and we have softened our phrasing in the Discussion; we no longer state there is 'no curative pharmacotherapy,' but rather that definitive, multisystem disease-modifying therapies are not yet established in routine practice.

		However, we maintain that our mapped operational pathway remains highly valid. As we now note in the revised Discussion, while emerging studies (such as the cited Annals trial) show promising pharmacological options, these often focus exclusively on single outcomes such as fatigue. Because they do not yet address the full, heterogeneous, multisystem presentation of Long COVID, patients still fundamentally require the symptom-led, multidisciplinary rehabilitation pathway mapped in our study. Nevertheless, we have added text explicitly acknowledging that as new pharmacological options gain evidence and enter formal guidelines, this pathway must remain dynamic and will likely necessitate future structural updates.<br><br>Revision<br>Discussion section (revised)<br>It is important to contextualise that at the time of data collection (mid-to-late 2024), definitive, disease-modifying pharmacotherapy was not yet widely established in routine NHS practice. While the evidence base is rapidly evolving—with recent clinical trials demonstrating promising targeted pharmacological interventions for specific symptoms such as fatigue [Ref: Annals]—these novel options currently address isolated outcomes rather than providing a holistic cure for the condition's complex, multisystem presentation. Therefore, the reliance on a rehabilitation-centred approach, coordinated multidisciplinary teamwork (MDT), and the careful monitoring of functional outcomes remains operationally valid and closely mirrors national guidelines [Zeraatkar]. However, as emerging pharmacological therapeutics mature and formally enter clinical commissioning pathways, the operational reality mapped in this study must remain dynamic. The introduction of definitive medical treatments will likely fundamentally alter the future GP-specialist dynamic, necessitating iterative updates to the care pathway.

7	The flowchart presents "Is Long COVID suspected?" as a binary YES/UNCERTAIN/NO decision despite the preceding theme emphasizing profound diagnostic uncertainty. Does the visualization adequately represent operational reality, or does it suggest deterministic decision-making?	**Response 7:** We have updated the flowchart legend (Figure 2) and the corresponding text to explicitly clarify that a "YES" or "Confirmed" node in the diagram does not represent biological certainty. Rather, it represents an administrative/pragmatic label that clinicians are forced to apply to a patient in order to unlock funding or trigger a referral to a specialist clinic. We highlight this as a core tension: the bureaucratic decision tree demands determinism, despite the underlying clinical ambiguity.<br><br>Revision<br>Result section<br><br>It is important to note that a 'Yes' or 'Confirmed' decision within this pathway does not represent biological certainty. Rather, it represents an administrative or pragmatic label that clinicians are compelled to apply to a patient to unlock funding or trigger a referral to a specialist clinic, highlighting the core tension between bureaucratic decision trees and underlying clinical ambiguity"<br><br>"Figure 2. Sequential Care Pathway for the Management of Long COVID. ... Note: Decision nodes (e.g., 'Confirmed as Long COVID?') represent administrative labels required to navigate clinical triage and unlock service funding, rather than absolute biological certainty".
8	The paper identifies systemic challenges but does not distinguish between barriers intrinsic to disease biology (lack of biomarkers) versus remediable through service design (lack of integration). For policymakers, this distinction is essential.	**Response 8**: We have added a dedicated paragraph to the Discussion explicitly separating these factors to aid policymakers. We clearly delineate that intrinsic biological barriers (the lack of a definitive biomarker and multisystem heterogeneity) drive diagnostic uncertainty, which cannot be immediately fixed by policy. In contrast, remediable service design barriers (such as siloed single-organ referral systems, the lack of funded MDT case conferences, and poor post-discharge digital tracking) are entirely within the control of local commissioners to optimise.

		Revision<br>Discussion(added)<br>“For policymakers and commissioners, it is essential to distinguish between challenges that are intrinsic to the disease biology and those that are remediable through service design. Intrinsic biological barriers—such as the lack of a definitive biomarker and multisystem heterogeneity—drive diagnostic uncertainty, which cannot be immediately resolved by health policy. In contrast, remediable service design barriers—such as siloed single-organ referral systems, the lack of funded MDT case conferences, and poor post-discharge digital tracking—are entirely within the control of local commissioners to optimise.”

VERSION 2 - REVIEW

Reviewer 1

Name Ramasawmy, Mel

Affiliation Queen Mary University of London, Institute of Health Informatics

Date 20-Aug-2026

COI

I commend the authors for their thorough and thoughtful approach to both reviewer suggestions. There is greater clarity in the methods and limitations. Adding context to the commissioning of services, changes over time, and the potential impact of emerging pharmacological evidence has emphasised the new insights this research contributes to understanding long COVID care, and strengthened messages on policy and practice implications.

Reviewer 2

Name Milic, Jovana

Affiliation University of Modena and Reggio Emilia Faculty of Medicine and Surgery

Date 15-Aug-2026

COI

The authors have addressed all the issues raised by the reviewers in great detail and have carefully considered and appreciated both reviewers' comments.
