# Supplementary material for: Exploring long covid care pathways in the UK NHS: a qualitative study of healthcare professionals’ experiences
Source: BMJ Open. 2026 Sep 15;16(9):e120704. doi: 10.1136/bmjopen-2026-120704 (PMC13583841; doi:10.1136/bmjopen-2026-120704)
Supplement: Supplementary file 2 [file bmjopen-16-9-s001.docx]

**Supplementary Materials**

**Title of Article:** Exploring Long COVID Care Pathways in the UK NHS: A Qualitative Study of Healthcare Professionals' Experiences.

**Supplementary File S1**. COREQ (Consolidated criteria for Reporting Qualitative research) Checklist

| Item No. | Guide Questions / Description | Reported Section / Details |
| --- | --- | --- |
| Domain 1: Research team and reflexivity |  |  |
| Personal characteristics |  |  |
| 1. Interviewer/facilitator | Which author/s conducted the interview or focus group? | Lead author (LPD) conducted the interviews. (*Methods – Data Collection*) |
| 2. Credentials | What were the researcher's credentials? (e.g. PhD, MD) | MBBS, MHPM, MPH, AFHEA, PhD candidate. (*Title Page*) |
| 3. Occupation | What was their occupation at the time of the study? | Academic researcher / PhD candidate. |
| 4. Gender | Was the researcher male or female? | The lead interviewer is male. |
| 5. Experience and training | What experience or training did the researcher have? | Training in qualitative methodologies and Reflexive Thematic Analysis (RTA). (*Methods – Reflexivity*) |
| Relationship with participants |  |  |
| 6. Relationship established | Was a relationship established prior to study commencement? | No prior relationship was established between the researchers and the participants. |
| 7. Participant knowledge of the interviewer | What did the participants know about the researcher? | Participants were provided with an information sheet explaining the researcher’s academic role and the study's aim to map Long COVID care pathways. (*Methods – Sampling and Recruitment*) |
| 8. Interviewer characteristics | What characteristics were reported about the interviewer/facilitator? | The researchers explicitly acknowledged their positionality as independent "outsiders" to the Croydon clinical teams, which helped minimise social desirability bias. (*Methods – Reflexivity*) |
| Domain 2: Study design |  |  |
| Theoretical framework |  |  |
| 9. Methodological Orientation and Theory | What methodological orientation was stated to underpin the study? | Interpretive-constructivist paradigm; Reflexive Thematic Analysis (RTA). (*Methods – Research Paradigm and Design*) |
| Participant selection |  |  |
| 10. Sampling | How were participants selected? | A two-stage purposive sampling strategy targeting clinical leads and core MDT disciplines. (*Methods – Participants and Recruitment*) |
| 11. Method of approach | How were participants approached? | Via targeted invitation emails. (*Methods – Participants and Recruitment*) |
| 12. Sample size | How many participants were in the study? | n = 12. (*Methods – Participants and Recruitment*) |
| 13. Non-participation | How many people refused to participate or dropped out? Reasons? | 15 clinicians were approached; 3 declined to participate due to severe clinical workloads. (*Methods – Sampling and Recruitment*) |
| Setting |  |  |
| 14. Setting of data collection | Where was the data collected? | Synchronous interviews were conducted face-to-face or via telephone; asynchronous interviews via secure, encrypted email. (*Methods – Data Collection*) |
| 15. Presence of non-participants | Was anyone else present besides the participants and researchers? | No. All synchronous interviews were conducted one-on-one. (*Methods – Sampling and Recruitment*) |
| 16. Description of sample | What are the important characteristics of the sample? | Professional background and years of clinical experience are detailed in the manuscript. (*Results – Table 1*) |
| Data collection |  |  |
| 17. Interview guide | Were questions, prompts, guides provided by the authors? Was it pilot tested? | A topic guide was developed from NICE NG188 guidance and piloted with one independent GP prior to formal use. (*Methods – Data Collection*) |
| 18. Repeat interviews | Were repeat interviews carried out? If yes, how many? | No repeat synchronous interviews. Email interviews utilised an iterative protocol involving 2–3 rounds of targeted follow-up exchanges. (*Methods – Data Collection*) |
| 19. Audio/visual recording | Did the research use audio or visual recording to collect the data? | Yes, synchronous interviews were audio-recorded. (*Methods – Data Collection*) |
| 20. Field notes | Were field notes made during and/or after the interview or focus group? | Yes, a reflexive journal and analytic memos were maintained throughout the study. (*Methods – Reflexivity*) |
| 21. Duration | What was the duration of the interviews or focus group? | Synchronous: 25–45 minutes. Asynchronous (Email): yielded a median of 800 words per participant. (*Methods – Data Collection*) |
| 22. Data saturation | Was data saturation discussed? | The "Information Power" framework (Malterud et al., 2016) was utilised to justify sampling sufficiency, rather than grounded-theory saturation. (*Methods – Sample Size Justification*) |
| 23. Transcripts returned | Were transcripts returned to participants for comment and/or correction? | No. Transcripts were not returned to avoid increasing the administrative burden on time-poor clinicians. (*Methods – Data Collection*) |
| Domain 3: Analysis and findings |  |  |
| Data analysis |  |  |
| 24. Number of data coders | How many data coders coded the data? | Lead researcher coded all data; a second researcher independently coded a subset (n=4) to foster reflexive dialogue. (*Methods – Analytical Procedure*) |
| 25. Description of the coding tree | Did authors provide a description of the coding tree? | Yes, an analytic audit trail illustrating the progression from raw data to final themes is provided. (*Supplementary Table S1*) |
| 26. Derivation of themes | Were themes identified in advance or derived from the data? | Themes were actively constructed inductively from the data using RTA. (*Methods – Analytical Procedure*) |
| 27. Software | What software, if applicable, was used to manage the data? | NVivo (Release 14, Lumivero, Denver, CO, USA). (*Methods – Analytical Procedure*) |
| 28. Participant checking | Did participants provide feedback on the findings? | Yes. A stakeholder sense-checking exercise was conducted with two participants and a service lead to ensure the pathway mapped accurately to operational reality. (*Methods – Analytical Procedure: Pathway Mapping*) |
| Reporting |  |  |
| 29. Quotations presented | Were participant quotations presented to illustrate the themes/findings? | Yes. Illustrative block quotes are provided, identified by Participant ID and Profession. (*Results*) |
| 30. Data and findings consistent | Was there consistency between the data presented and the findings? | Yes. The derivation of themes from data is explicitly mapped in Table 3 and Supplementary Table S1. (*Results*) |
| 31. Clarity of major themes | Were major themes clearly presented in the findings? | Yes. Four central analytical themes are presented. (*Results*) |
| 32. Clarity of minor themes | Is there a description of diverse cases or a discussion of minor themes? | Yes. Key concepts/subthemes and differing professional perspectives (e.g., GP vs. Consultant) are discussed. (*Results & Table 3*) |

**Supplementary File S2**. Interview Guide related to mapping out the long COVID care pathway and healthcare professionals’ experience and practices

| No | Question |  |
| --- | --- | --- |
| **Section A: Basic Information** | | |
| 1. | Gender |  |
| 2. | Could you please tell me about your professional background and area of expertise?  **Responses :** | Profession: Doctor or Nurse, Physiotherapist etc  Consultant, Speciality Registrar  Your specialities: Chest/respiratory, cardiology, GIT etc |
| 3. | Could you please share your year of experience in practice?  **Responses :** | Years of experience |
| 4. | What was your experience using care pathways in patient management?  **Responses :** | Clinical Care pathways, in general, not limited to Long COVID |
| **SECTION: Symptoms and Sign** | | |
| 5. | What are the most common symptoms and clinical signs seen in those individuals with long COVID?  **Responses :** | Symptoms and signs in long COVID patients |
| 6. | Do you use specific criteria to determine the severity of the disease before choosing a treatment option?  **Responses :** | How do you classify long COVID cases into mild, moderate, or severe? Does such a classification exist? Is any other classification used? Do you base the management option on the severity of the disease? |
| **Section: Your Role** | | |
| 7. | What is your specific role in managing patients with long COVID in this health facility?  **Responses :** | In what ways are you involved in the care of long COVID patients at this facility?  How do you collaborate with other healthcare professionals in managing patients with long COVID at this facility? |
| 8. | Based on your role, do you assess (diagnose) patients for long COVID?  **Responses :** | How do you determine if a patient may be experiencing symptoms of long COVID?  What steps do you take to ensure accurate diagnosis of long COVID in patients?  In your experience, what types of patients are most at risk for developing long COVID?  Are there any specific challenges you have encountered in diagnosing or treating patients with long COVID? |
| **Section: Tool** | | |
| 9. | Do you have a specific clinical tool or criteria for diagnosing this new disease, or do you rely on your clinical knowledge and experience?  **Responses :** |  |
| 10. | Who is responsible for evaluating patients with long COVID, given the various tools different healthcare professionals use?  **Responses :** | Different long COVID assessment tools were observed; who is responsible for confirming the diagnosis? |
| **Referral** | | |
| 11. | When referring a suspected long COVID patient, who is the first healthcare professional to evaluate a long COVID patient?  **Responses :** | Who do you send a patient with suspected long COVID to for further evaluation? |
| 12. | Who is the next person the patient will see after the diagnosis is made, and what will determine who the patient will see next?  **Responses :** | After you have confirmed the diagnosis, who do you refer or where for further management? |
| 13. | Who is responsible for discharging the patient home from the clinic or rehabilitation centre? Is the patient discharged home or to other health care facilities? What determines where to refer this patient or who sees the patient next?  **Responses :** | Who discharges patients' home or to homecare from the clinic and rehabilitation centre? |
| **Treatment and Continue Care** | | |
| 14. | What are the available treatment options for patients, and how do you determine the most appropriate option for each patient?  **Responses :** | How do you manage long COVID patients? Why do you choose such management options? |
| 15. | How do you ensure patient continuity of care, especially when transitioning between different healthcare settings?  **Responses :** | Do you follow the patient up? And how? |
| 16. | How do you assess patients' progress during treatment, and do you still follow up with them after they are discharged?  **Responses :** | How do you assess the patient's progress during management? |
| 17. | List the medical team you interact with when managing the patient  **Responses :** | Who are the other healthcare providers you interact with during the management of long COVID patients? |
| Other information | | |
| 18. | Is there any other information that you would like to share with me on the topic?  **Responses :** |  |
| 19. | Would you like to receive a summary of the findings of this study?  **Responses :** |  |

Thank you for your time.

**Supplementary File S3**.Participant characteristics

| Participant ID | Professional Background | Years of Clinical Experience (Range) |
| --- | --- | --- |
| P1 | General Practitioner (GP) | > 20 years |
| P2 | General Practitioner (GP) | 10 – 20 years |
| P3 | Respiratory Consultant | 10 – 20 years |
| P4 | General Practitioner (GP) | > 20 years |
| P5 | General Practitioner (GP) | > 20 years |
| P6 | Respiratory Consultant | > 20 years |
| P7 | Clinical Psychologist | < 10 years |
| P8 | Respiratory Physiotherapist | < 10 years |
| P9 | Specialist Nurse | 10 – 20 years |
| P10 | General Practitioner (GP) | 10 – 20 years |
| P11 | Specialist Nurse | 10 – 20 years |
| P12 | Specialist Nurse | 10 – 20 years |

**Supplementary File S4.** Summary of themes identified from healthcare professionals’ accounts of long COVID care

| Theme | Subtheme / Key Concept | Illustrative Quote |
| --- | --- | --- |
| 1. Navigating diagnostic uncertainty and subjective severity classification | Diagnosis based on symptom persistence and exclusion of alternative conditions | *"There is no definitive diagnostic test for long COVID… it is largely a diagnosis of exclusion based on persistent symptoms."* (P2, GP) |
|  | Reliance on clinician judgement to determine severity | *"We assess how much the symptoms are affecting their daily function, work and overall quality of life."* (P4, GP) |
|  | Variability in symptom presentation complicates assessment | *"Patients present with a wide range of symptoms and combinations, which makes classification and management quite complex."* (P6, Specialist Nurse) |
| 2. The General Practitioner as linchpin in fragmented referral pathways | GP as gatekeeper to specialist services | *"The majority are manageable in general practice… but in the absence of a clear, commissioned holistic long COVID service, referral might be made to respiratory, neurological or cardiovascular teams depending on the clinical picture."* (P1, GP) |
|  | Symptom-driven referral across multiple specialties | *"Patients with respiratory symptoms tend to go to the respiratory clinic, while fatigue or deconditioning cases are usually directed towards rehabilitation services."* (P3, GP) |
|  | Internal triage within specialist services | *"If they needed psychology, they would be referred to me, or if they needed physio, they would be referred to the physiotherapist."* (P7, Clinical Psychologist) |
| 3. Therapeutic pragmatism and symptom-led support | Individualised and negotiated care planning | *"Determining the most appropriate option is through discussion and negotiation with the patient… depending on the symptomatology and the patient."* (P1, GP) |
|  | Rehabilitation-focused interventions | *"If it was physiotherapy, it would be a home exercise programme or they would join the exercise classes… if it was occupational therapy it would be education on fatigue management."* (P8, Respiratory Physiotherapist) |
|  | Multidisciplinary but operationally segmented care | *"Different professionals take the lead depending on the main symptoms the patient presents with."* (P6, Specialist Nurse) |
| 4. The continuity gap and variable discharge practices | Reliance on patient-initiated follow-up in primary care | *"We often rely on patient-initiated contact. I can review long COVID symptoms when assessing other conditions."* (P5, GP) |
|  | Rehabilitation programmes with defined discharge points | *"Patients stay 6–12 months in the rehab before being discharged… we reassess them at the end to see if they have had improvement."* (P8, Respiratory Physiotherapist) |
|  | Limited post-discharge monitoring | *"No — we do not follow the patient that we send and discharge to the GP."* (P8, Respiratory Physiotherapist) |

**Supplementary File S5.** An analytic audit trail demonstrating the progression from raw data to a mapped pathway.

| Raw Data Extract | Initial Code | Candidate Theme | Final RTA Theme | Informed Pathway Node (Fig 2) |
| --- | --- | --- | --- | --- |
| *"I classify them by clinical severity and duration. I am not aware of verified scales." (P1, GP)* | No formal tools used | Subjective clinical assessment | Navigating diagnostic uncertainty | Step 2: Initial Evaluation |
| *"The symptoms overlap with many other diseases, and I try to exclude these before referral... arrange blood tests, ECG, CXR." (P10, GP)* | Ruling out other pathologies | Exclusionary diagnosis | Navigating diagnostic uncertainty | Step 3: Diagnostic Path |
| *"In the absence of a holistic service, referral might be made to respiratory, neurological or cardiovascular teams depending on the clinical picture." (P1, GP)* | Fragmented specialist routing | GP as gatekeeper | The GP as linchpin in fragmented pathways | Step 4: Referral Decision |
| *"If it was physiotherapy... home exercise. If psychology... for the brain fog." (P8, Physio)* | Segmented therapy roles | Multidisciplinary working | Therapeutic pragmatism & symptom-led support | Step 6: Management Planning |
| *"No, we do not follow the patient that we send and discharge to the GP, and we do not follow up again." (P8, Physio)* | Lack of post-discharge tracking | Patient-initiated follow-up | The continuity gap and variable discharge practices | Step 8: Discharge Planning |

**Supplementary File 6.** Transferable Insights: Linking Qualitative Themes to International Service Design Levers

| Qualitative theme | Transferable concept | Service design lever |
| --- | --- | --- |
| Navigating diagnostic uncertainty | Operationalising subjective severity | Triage design: Implementation of standardised, digitally integrated functional screening tools prior to specialist referral. |
| The GP as linchpin in fragmented pathways | The “gatekeeper burden” | Referral governance: Transition from single-organ referral models to centralised “hub-and-spoke” interdisciplinary triage systems. |
| Therapeutic pragmatism | Segmented versus integrated care | MDT integration: Implementation of shared electronic health records and funded, scheduled cross-speciality case conferences. |
| The continuity gap | Vulnerabilities in patient-initiated follow-up | Post-discharge governance: Establishment of automated digital follow-up mechanisms to monitor patient trajectories and prevent relapse. |

Note: Service design levers represent transferable organisational strategies derived from qualitative themes identified in interviews with healthcare professionals involved in long COVID care.
